# Indications and experience with counterclockwise rotation of the maxilla- mandibular complex

**DOI:** 10.1186/1746-160X-10-S1-O8

**Published:** 2014-12-12

**Authors:** Oliver Ploder

**Affiliations:** 1 Oral and Maxillofacial Unit, Academic Teaching Hospital Feldkirch, Austria

## 

In orthognathic treatment planning for the correction of dentofacial deformities imaging and planning are important steps prior to surgery. Based on the natural head orientation, the anterio-posterior and vertical position of the upper incisor tip is one of the key elements in aesthetic planning. Gum exposure and facial and smile harmony are factors that influence this position. After definition of the upper incisor tip position, the maxillomandibular complex (MMC) can be altered according to the treatment needs (severe class II deficiency, reduced airway space etc.).

Depending on the pivot point and the amount of rotation of the MMC, the hard and soft tissue changes can vary. Placement of the pivot point anteriorly (anterior nasal spine or upper incisor tip) results in an increase of the posterior face height and in extensive advancement of the lower face (up to 25 mm). In contrast, placement of the pivot point posteriorly reduces the anterior face height and has limited effect to the lower face profile. The advancement of the lower face is influenced by the location of the pivot point, the amount of rotation of the MMC (up to 5 degree), the amount of overjet corrected with sagittal split osteotomy and the amount of advancement of genioplasty.

Careful analysis of the cephalograms and the face and implementation of these data into the planning are important to optimize occlusal function and facial aesthetics after orthognathic surgery. The concept of rotation of the MMC in either way widens the spectrum in treatment planning.

